# Supplementary material for: Characterisation of the Historic Demographic Decline of the British European Polecat Population
Source: Mol Ecol. 2025 Sep 4;34(20):e70091. doi: 10.1111/mec.70091 (PMC12530300; doi:10.1111/mec.70091)
Supplement: Supplementary file 3 — Figure S1. Kinship coefficients between each of the 65 polecat and 8 domestic ferret individuals. KING estimates a relatedness‐PHI score which can range from > 0.354, [0.177, 0.354], [0.0884, 0.177] and [0.0442, 0.0884] and corresponds to duplicate/MZ twin, 1st‐degree, 2nd‐degree, and 3rd‐degree relationships respectively. Starred individuals (S17 & S0012_VWT665) were identified as having a 3rd‐degree relationship (0.076) and S17 was removed from further analyses. Figure S2. Percentage of variance explained by PC's 1–20 using whole‐genome SNPs. Figure S3. Average K value for replicated runs of ancestry for K 1–12. Figure S4. Isolation by distance of British samples plotted using a pairwise Hamming genetic distance and geographic distance matrix. Results revealed a positive correlation between genetic distance and geographic distance (r = 0.333, p = 0.001). Figure S5. Pairwise F st values for whole‐genome SNPs between populations. Figure S6. Ancestry proportions for each individual inferred from ADMIXTURE for K values of 2–5. Figure S7. Maximum likelihood phylogeny with 100 bootstrap replicates for genome‐wide SNPs from 64 samples. The number at nodes refer to bootstrap values of 95 and above. Branch lengths are in expected substitutions per site. Figure S8. Inbreeding estimates and heterozygosity comparisons across British and European populations (a) Population‐level inbreeding coefficient values (b) Proportion of heterozygous sites calculated using VCFtools and (c) Mean FROH per population. Figure S9. Estimated mean effective population size (solid line) with 95% confidence intervals (shaded area) for the Italian polecat population across 200 generations (generations = 4 years) based on whole‐genome SNP data computed in GONE. [file MEC-34-e70091-s003.docx]

**Supplemental Information for:**

**Characterisation of the historic demographic decline of the British European polecat population**

R. Shaw ^1,2^, J. MacPherson ^3^, A, C. Kitchener^4^, G, J. Etherington^1^, W. Haerty ^1,2^

^1^ Earlham Institute, Norwich Research Park, Colney Lane, Norwich, NR4 7UZ rebecca.shaw@earlham.ac.uk

^2^ University of East Anglia, Norwich Research Park, Norwich, NR4 7TJ

^3^ Vincent Wildlife Trust, Ledbury, Herefordshire, HR8 1EP

^4^ Department of Natural Sciences, National Museums Scotland, Edinburgh EH1 1JF, UKUK and School of Geosciences, University of Edinburgh, Drummond Street, Edinburgh EH8 9XP, UK

**Table of Contents:**

| **Figure S1** | Page 2 |
| --- | --- |
| **Figure S2** | Page 3 |
| **Figure S3** | Page 4 |
| **Figure S4** | Page 5 |
| **Figure S5** | Page 6 |
| **Figure S6** | Page 7 |
| **Figure S7** | Page 8 |
| **Figure S8** | Page 9 |
| **Figure S9** | Page 10 |


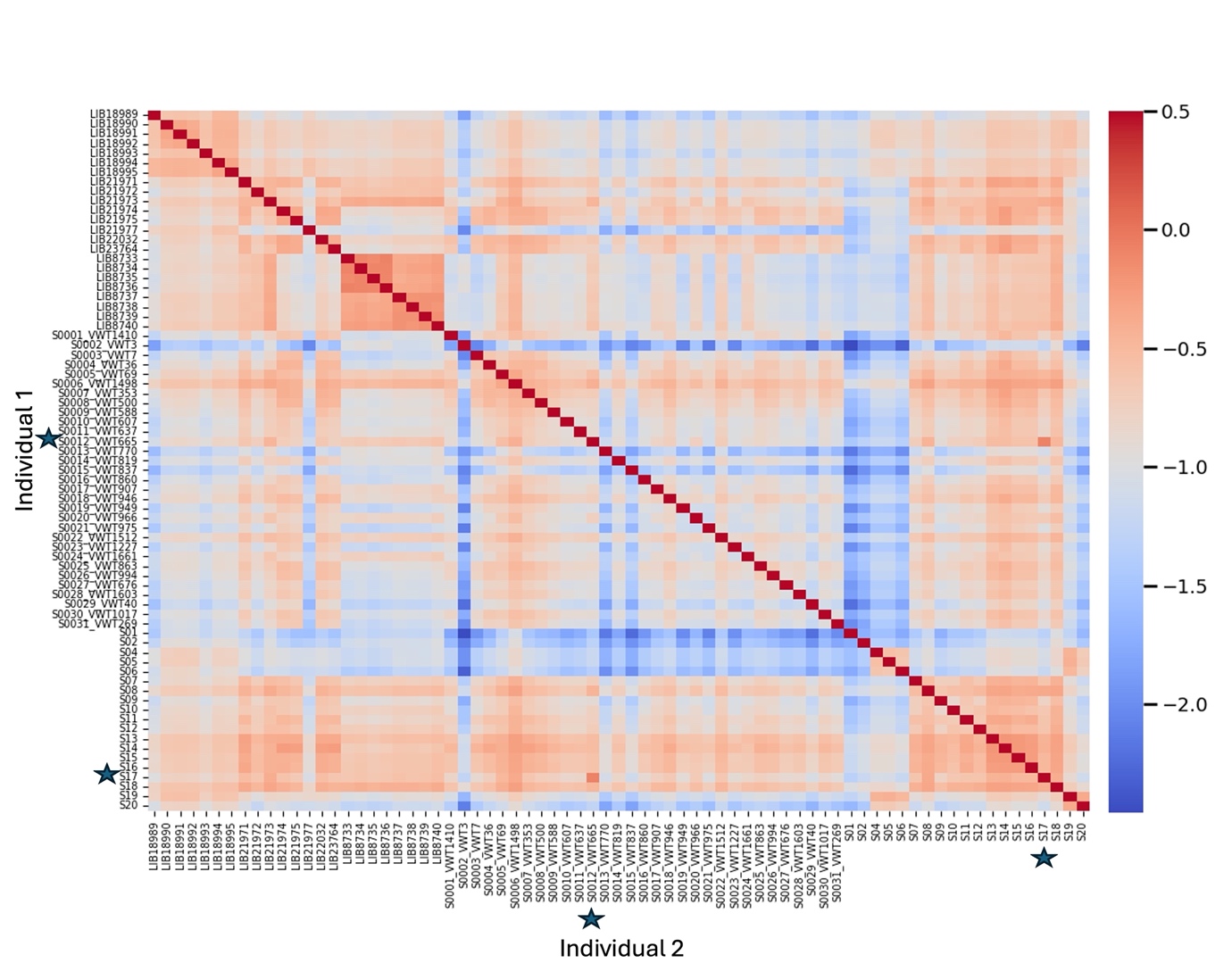


**Figure S1**: Kinship coefficients between each of the 65 polecat and 8 domestic ferret individuals. KING estimates a relatedness-PHI score which can range from >0.354, [0.177, 0.354], [0.0884, 0.177] and [0.0442, 0.0884] and corresponds to duplicate/MZ twin, 1st-degree, 2nd-degree, and 3rd-degree relationships respectively. Starred individuals (S17 & S0012_VWT665) were identified as having a 3rd-degree relationship (0.076) and S17 was removed from further analyses.


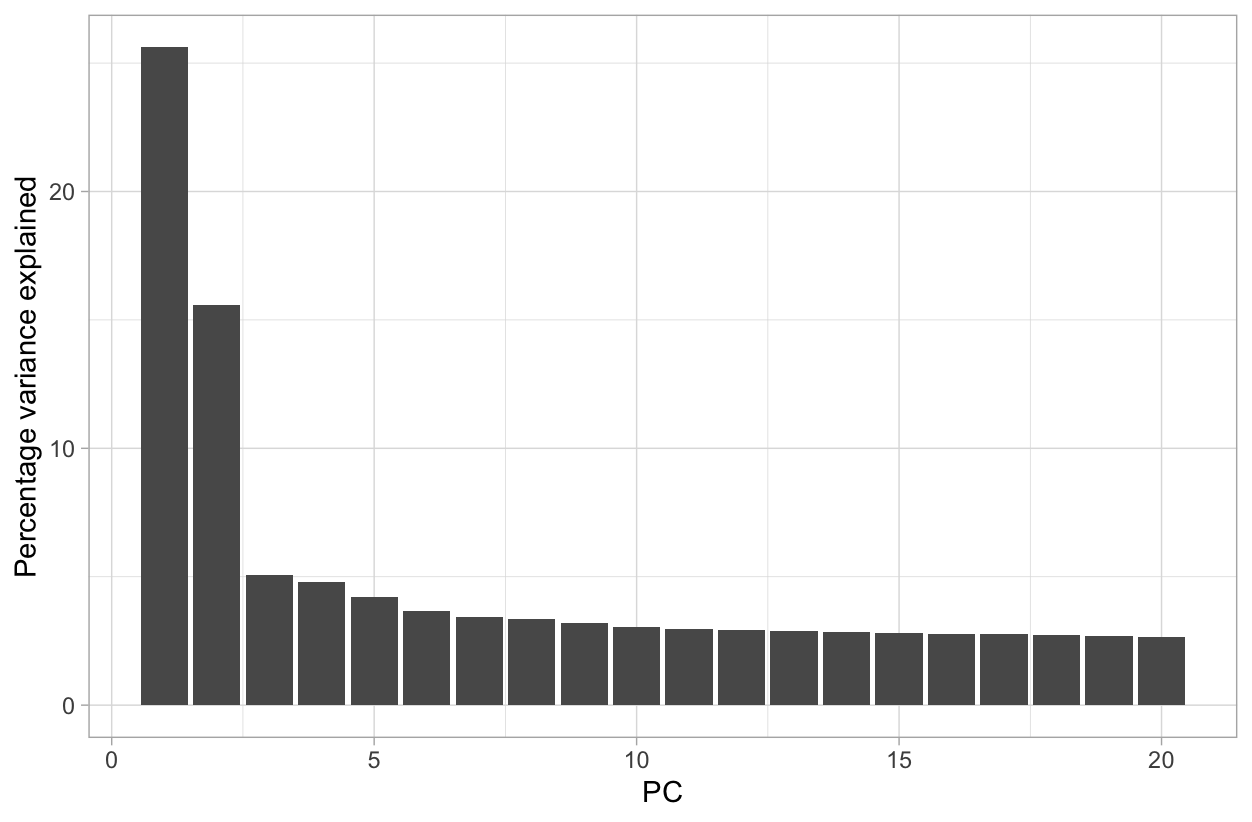


**Figure S2**: Percentage of variance explained by PC’s 1-20 using whole-genome SNPs.


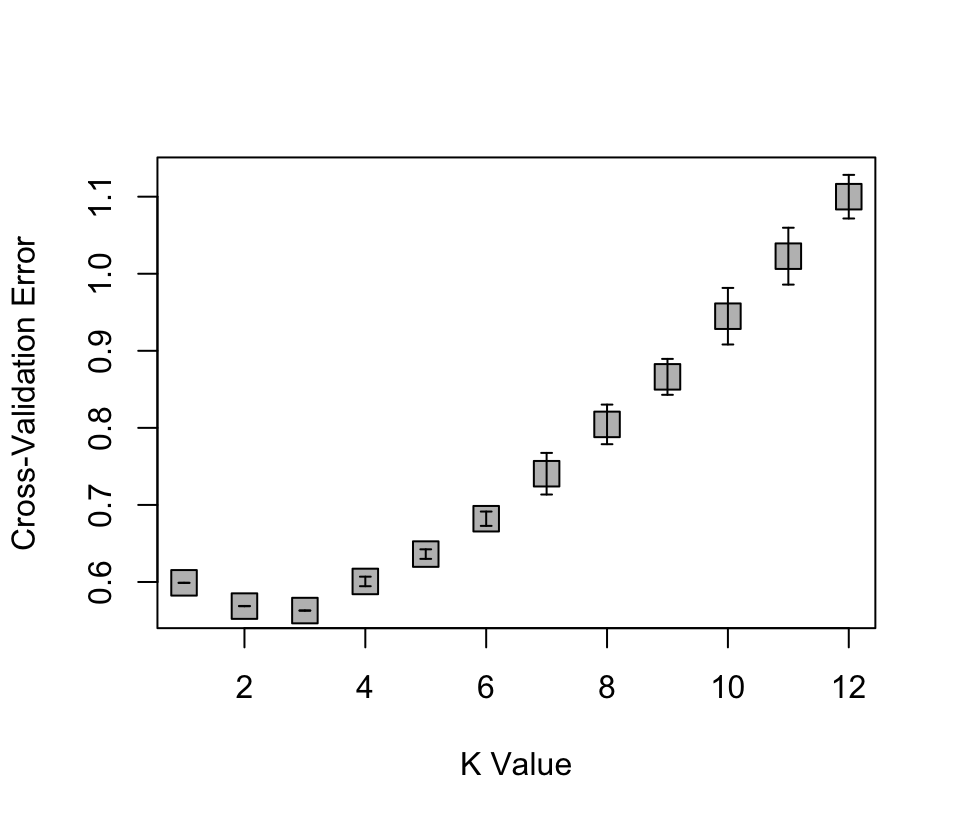


**Figure S3:** Average K value for replicated runs of ancestry for K 1-12**.**


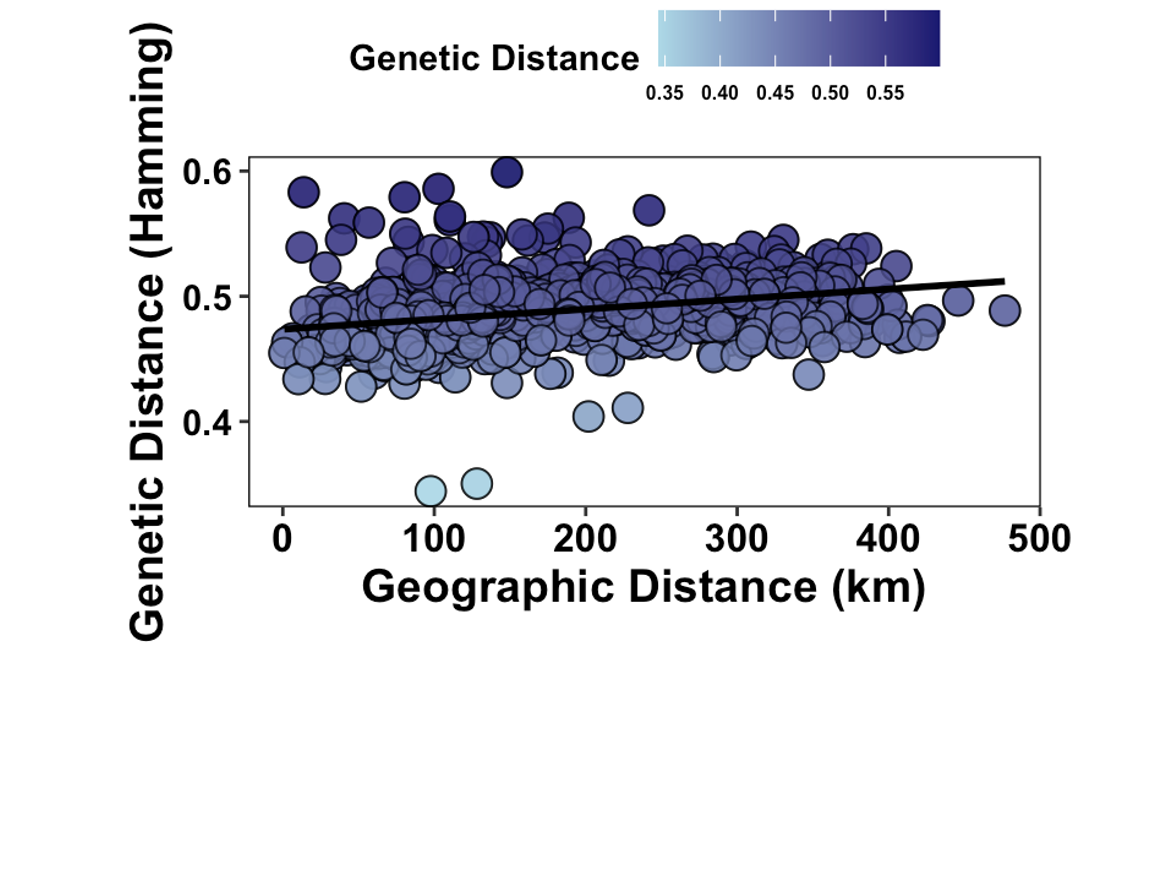


**Figure S4**: Isolation by distance of British samples plotted using a pairwise Hamming genetic distance and geographic distance matrix. Results revealed a positive correlation between genetic distance and geographic distance (*r = 0.333, p = 0.001*).


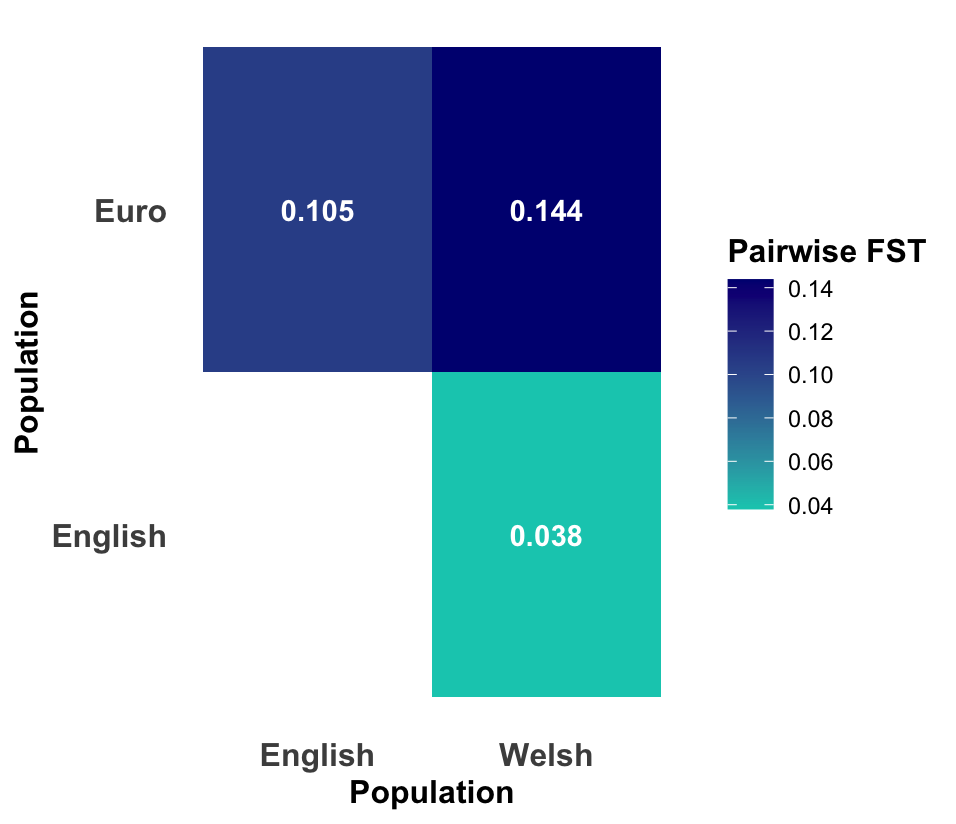


**Figure S5**: Pairwise F_ST_ values for whole genome SNPs between populations.

**
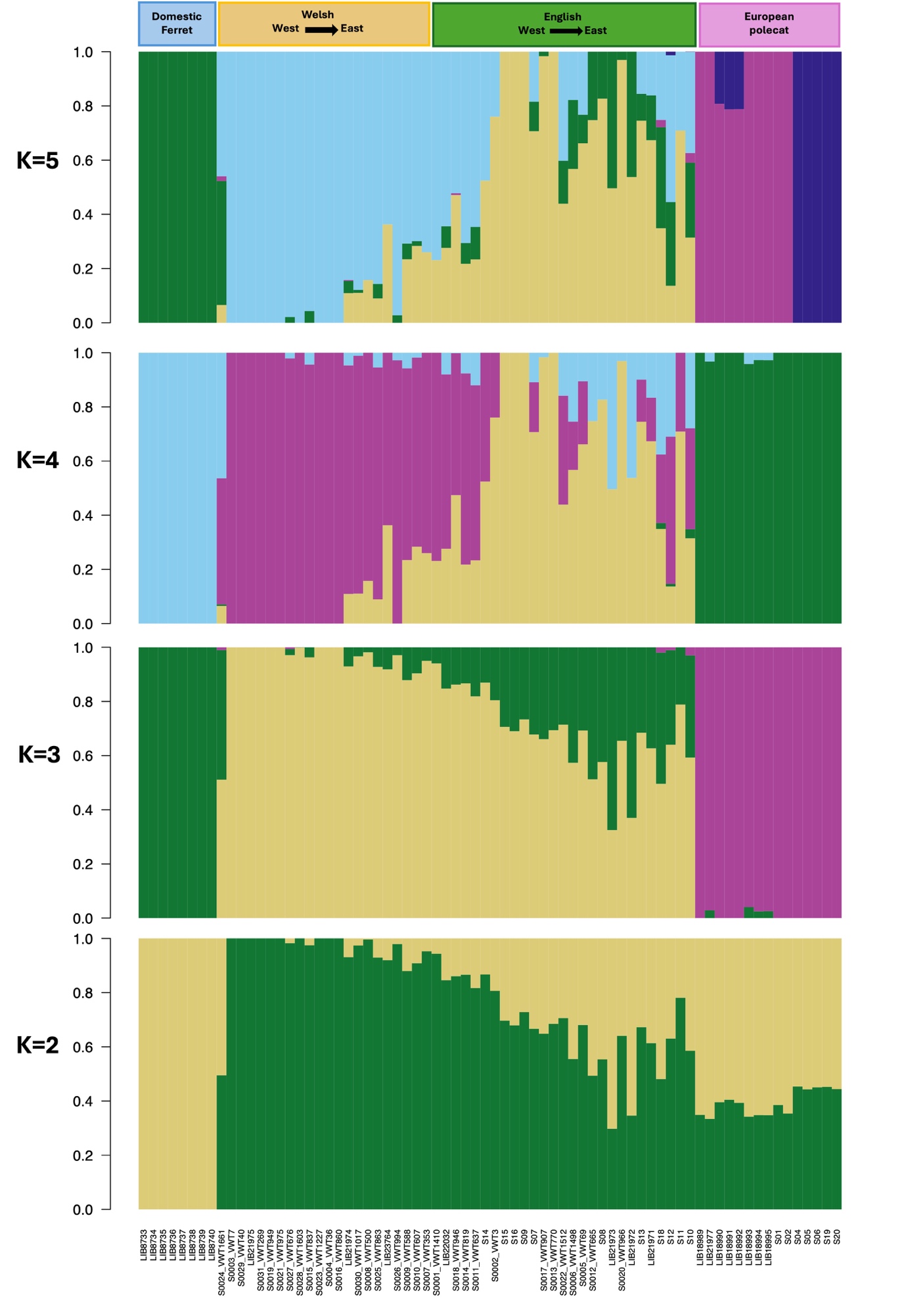
Figure S6:** Ancestry proportions for each individual inferred from ADMIXTURE for K values of 2-5.


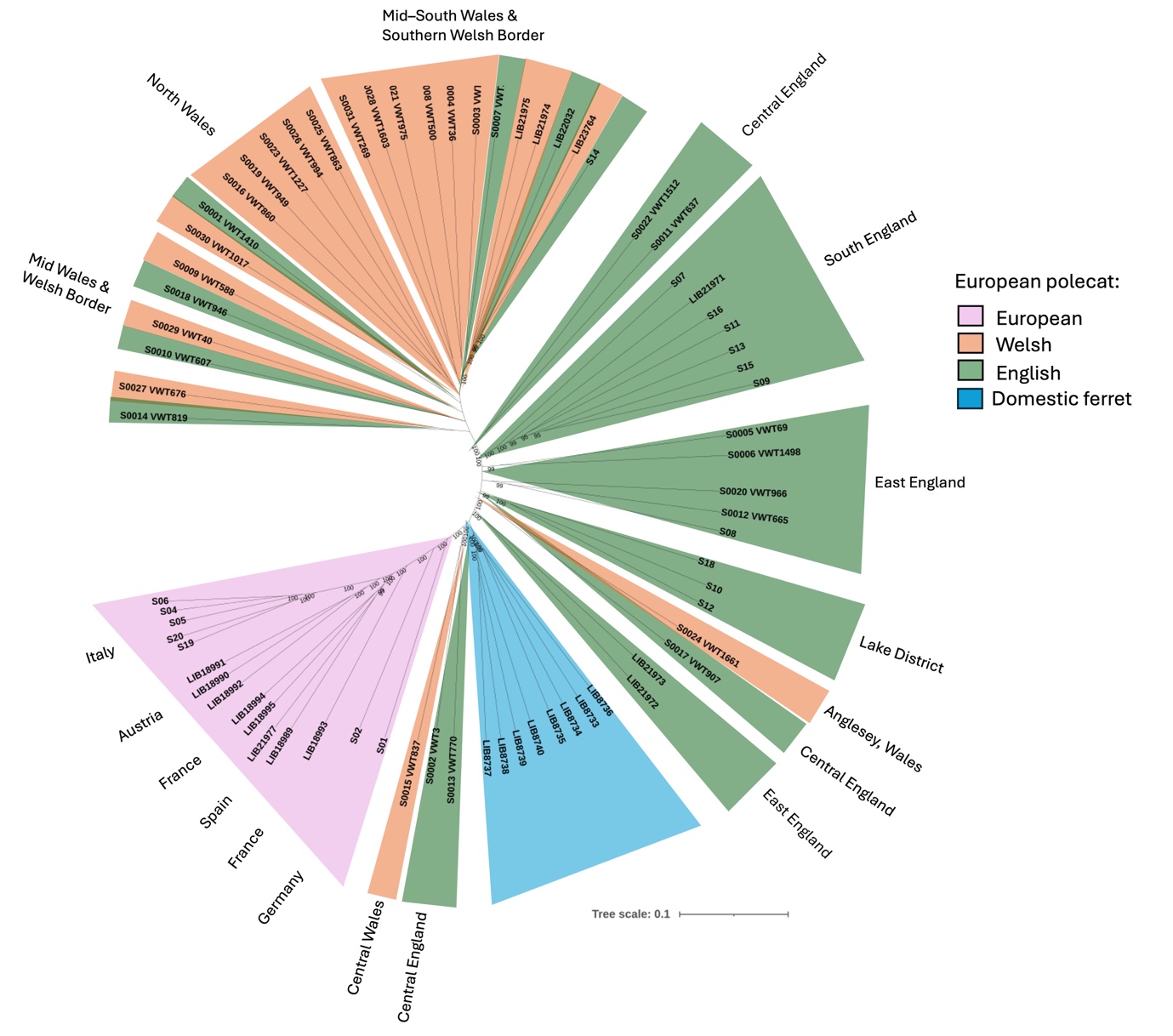


**Figure S7**: Maximum likelihood phylogeny with 100 bootstrap replicates for genome-wide SNPs from 64 samples. The number at nodes refer to bootstrap values of 95 and above. Branch lengths are in expected substitutions per site.


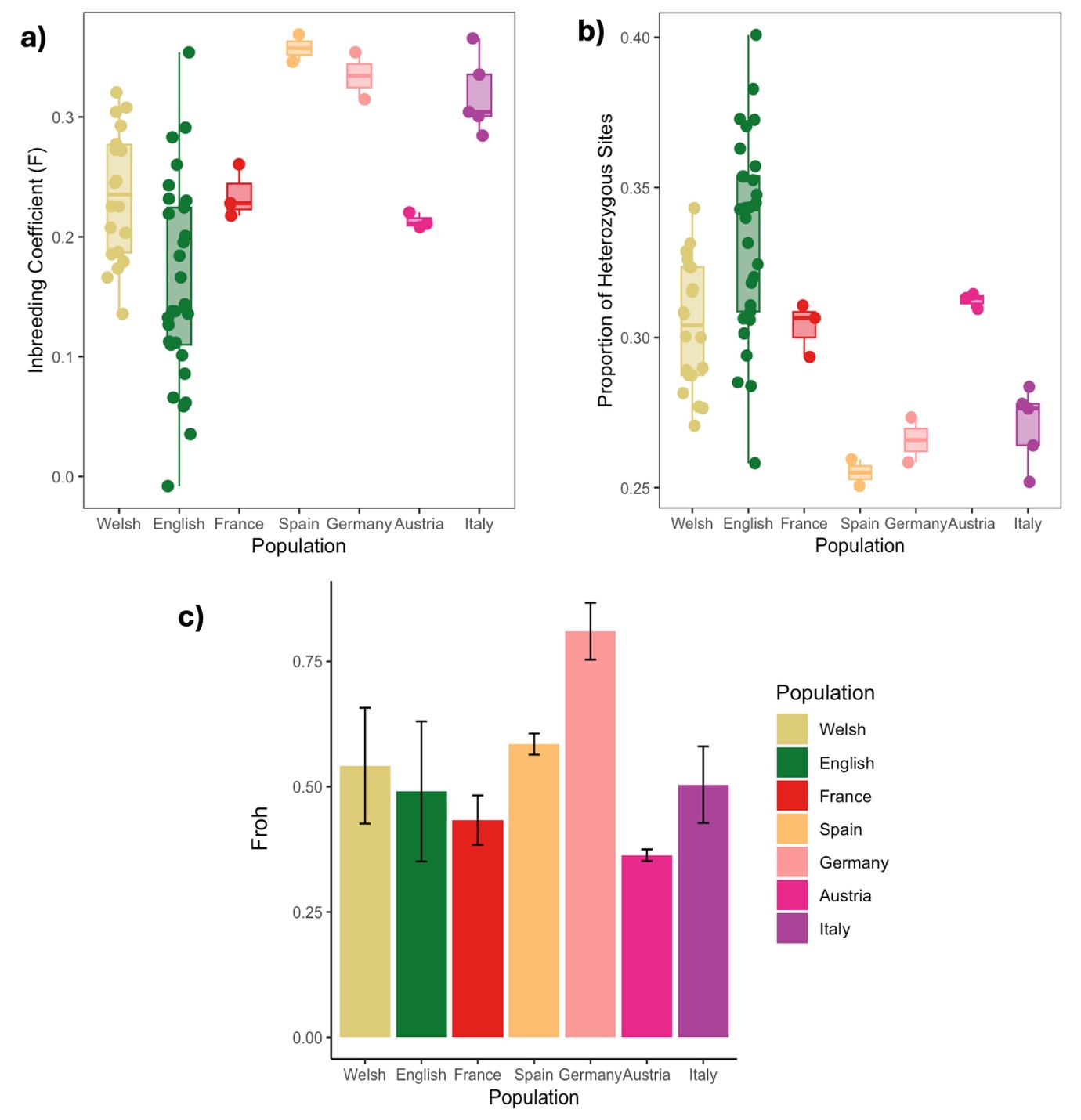


**Figure S8**: Inbreeding estimates and heterozygosity comparisons across British and European populations (a) Population-level inbreeding coefficient values (b) Proportion of heterozygous sites calculated using VCFtools and (c) Mean F_ROH_ per population


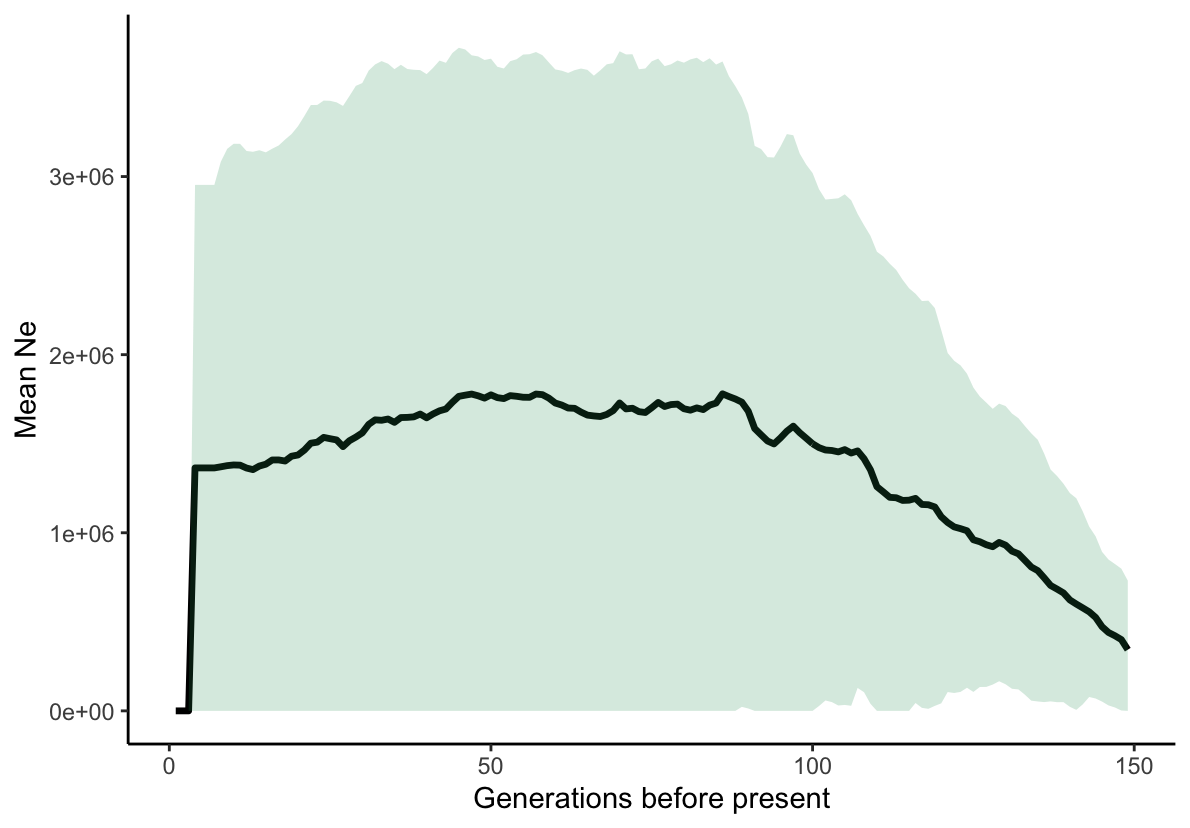


**Figure S9:** Estimated mean effective population size (solid line) with 95% confidence intervals (shaded area) for the Italian polecat population across 200 generations (generations = 4 years) based on whole genome SNP data computed in GONE.
